# Aligning labour force perceptions to job attraction, satisfaction and retention strategies in horticulture: A data-driven empirical study for Australian banana industry

**DOI:** 10.1371/journal.pone.0344799

**Published:** 2026-04-20

**Authors:** Mallika Roy, Delwar Akbar, Darshana Rajapaksa, Azad Rahman

**Affiliations:** 1 School of Business and Law, Central Queensland University, Norman Gardens, Queensland, Australia; 2 Department of Economics, University of Chittagong, Address: Chittagong University Rd, Chittagong, Bangladesh; 3 Department of the Environment, Tourism, Science and Innovation, Queensland Government, Brisbane, Australia; 4 School of Engineering and Technology, Central Queensland University, Norman Gardens, Queensland, Australia; Guangxi Normal University, CHINA

## Abstract

Workforce stability in the banana industry is critical for productivity, fruit quality, and long-term sustainability. Labour shortages and high turnover can also lead to harvesting delays and handling inefficiencies, creating significant food waste in horticultural supply chains. Despite increasing policy attention, limited research has explored how job-related factors, workplace ergonomics, and labour force perceptions influence motivation, satisfaction, and retention. This study addresses that gap by examining key socio-demographic and perceptual drivers affecting labour dynamics in the Australian banana industry. Using a survey of 1,202 participants across 10 urban and regional locations in Queensland, the study applies Economic Psychology and behavioural economics to explore how satisfaction, perception, and aspiration biases interact with physical work conditions. Q-Q plots and ordinal regression analysis reveal that training and development (β = 0.35, p < 0.01), indicating a strong positive effect on job satisfaction, ergonomic workplace design (β = 0.29, p < 0.01), suggesting improved physical comfort enhances satisfaction, and salary equity (β = 0.22, p < 0.05), reflecting the motivational role of perceived pay fairness, significantly enhance job satisfaction. Retention is strongly influenced by growth opportunities (β = 0.38, p < 0.01), implying that career progression reduces turnover intentions, and perceived industry stability (β = 0.31, p < 0.05), highlighting the importance of long-term employment confidence. These findings highlight the need for ergonomically supportive, employee-focused practices to align with workforce expectations. The study offers practical insights for stakeholders seeking to improve labour sustainability, reduce turnover, and enhance resilience within Australia’s agricultural supply chains.

## 1. Introduction

Research on horticultural labour broadly converges around three interrelated themes: persistent workforce shortages, challenges in labour retention and satisfaction, and the implications of these issues for productivity and sustainability. Globally, the horticulture sector plays a vital role in food security and economic development [1,2]. Yet it faces persistent workforce challenges, including labour shortages, high turnover, and inconsistent employment conditions [2–4]. These challenges increasingly threaten productivity, intensify agri-food waste, as labour shortages and high turnover lead to delayed harvesting, improper handling, and inefficient postharvest practices that increase spoilage and resource loss, product quality, and long-term sustainability across agricultural supply chains [5–7]. Rather than isolated problems, these challenges interact across agricultural supply chains, directly influencing output quality, operational efficiency, and long-term sectoral resilience [8–10].

Within this global landscape, the Australian banana industry—largely concentrated in Queensland—represents a critical yet underexamined case [11–13]. The industry supports both domestic consumption and export markets and has experienced growing demand in recent years [13–15]. However, this expansion has intensified pressure on labour availability, particularly for physically demanding and seasonal farm work [16,17,18]. As a result, attracting and retaining a stable and skilled workforce has emerged as a critical constraint on industry performance [19,18]. While demand for bananas has expanded, existing research largely frames labour issues as a supply constraint, paying limited attention to the lived work experience of employees. Consequently, workforce discussions often remain detached from job design, physical work demands, and employee expectations, despite their relevance for retention.

A second stream of literature focuses on job satisfaction, motivation, and retention in agricultural and rural employment. Given the horticulture industry’s dependence including banana industry on a reliable workforce, understanding the factors that influence job attraction, satisfaction, and retention is essential for its long-term sustainability [3,20]. Evidence from agricultural and rural employment studies suggests that high physical and psychological job demands can negatively affect workplace satisfaction, safety, and retention, underscoring the importance of work design, training, and employee support mechanisms in labour-intensive industries [15,21].

A third body of research examines labour dynamics using broad or sector-agnostic indicators, often prioritising production efficiency or land-use outcomes over workforce perceptions. Existing studies often generalise findings across agricultural sectors or focus primarily on production and land-use dynamics, providing only partial insight into workforce behaviour [22,23,18]. Moreover, existing research tends to apply broad measures of satisfaction without considering industry-specific elements like work conditions, benefits, or job appeal relative to other sectors [24,25]. Additionally, many studies rely on basic statistical techniques and overlook ergonomic working conditions, perceived fairness, and comparative job appeal. The limited integration of perceptual factors constrains the explanatory power of existing workforce models in horticulture.

This study addresses these gaps by systematically examining how ergonomic work conditions, labour perceptions, and expectations influence job attraction, satisfaction, and retention in Australia’s banana industry. Drawing on a large-scale survey and applying ordinal regression analysis, the study identifies key socio-demographic and perceptual drivers of labour dynamics. By integrating workforce perceptions with ergonomic and behavioural considerations, the study offers actionable insights for policy development, human resource management, and workplace design aimed at improving labour sustainability and reducing turnover in agricultural supply chains.

## 2. Literature review

There are numerous factors within a job—such as fair wages, job security, supportive work environments, and growth opportunities—that significantly influence labour force attraction to and retention in a role. Parmer et al. demonstrated that employees who perceive their organization as having a strong stakeholder orientation report higher job satisfaction, with employee perspective-taking acting as a potential mediator, thus highlighting the cognitive and attitudinal impacts of stakeholder-oriented values [26]. Similarly, Dawkins et al., using the Theory of Planned Behaviour, found that while CSR perceptions influenced job choice intentions across individuals, the contributing factors—attitudes, subjective norms, and perceived behavioural control—differed by culture, stressing the need to tailor CSR and recruitment strategies to local contexts [27]. Gender was also found to moderate the relationship between job demands and job satisfaction, suggesting that men and women may perceive and respond to workplace demands and autonomy differently [28]. Drawing on signalling and social identity theories, Greening and Turban found that corporate social performance (CSP) positively affects job pursuit intentions by signalling a favourable work environment and enhancing applicants’ self-image, with experimental evidence indicating a preference for socially responsible firms [29].

The horticulture sector, especially the banana industry, is a critical component of agricultural economies worldwide but faces persistent workforce challenges [30,31]. Labour shortages, high turnover, and dependence on temporary or migrant labour are common issues undermining productivity and long-term sustainability [3,32,33]. Addressing these issues requires understanding what drives job motivation, satisfaction, and retention in this sector. Prior studies have highlighted job satisfaction and retention as key to workforce stability [24,34,35], with factors such as wages, working conditions, and career development playing a central role [36,37]. Cahill et al. emphasized that macroeconomic changes can significantly influence job satisfaction, engagement, and work–life balance [38], while Mohanty linked job satisfaction to wage and working hours, with variations across age groups [39]. Tran highlighted how personality traits such as internal locus of control positively affect job satisfaction via more favourable job perceptions [40], and Poggi argued that unmet aspirations reduce satisfaction more than meeting basic expectations increases it [41]. Taylor and Bisson stressed the urgency of training older workers to address the expected skilled labour shortage, advocating for age-inclusive learning theories [42]. Crossley and Highhouse found that deliberate job search strategies lead to greater satisfaction than intuitive methods, particularly among future-oriented individuals [43]. The study by Froehlich et al. explored that while chronological age alone is a poor predictor of employability, it indirectly influences employability through mediating factors such as employees’ motivation (future time perspective and goal orientation) and engagement in formal and informal learning activities [44]. A study by Staelens et al. revealed that job satisfaction in the agriculture supply chain is significantly influenced by extrinsic rewards such as wages, job security, and a healthy work environment, with dissatisfaction and access to alternative livelihoods increasing the intention to leave, while participation in informal workplace savings networks reduces this intention [35].

Job factors emerge as a critical link between labour dynamics and agri-food waste [45,46] Low wages, seasonal instability, and limited career progression [47,48] weaken human capital investment and drive high turnover, which in turn causes harvesting delays, handling errors, and postharvest losses. Circular economy practices and smart-farming technologies—such as IoT, AI, and blockchain—require stable, skilled labour to implement closed-loop systems, precision harvesting, and efficient resource tracking [49–51]. Integrating better wages, secure employment, career pathways, and training with these green supply chain and agri-tech innovations therefore strengthens workforce capacity, enabling the reduction of food waste and advancing circular economy goals across horticultural supply chains [52,53].

Despite the broad literature, limited research has examined these dynamics specifically within the Australian horticulture or banana sectors [25,54,55]. Understanding industry-specific factors can guide retention strategies and improve workforce sustainability [35,56]. Although global studies—from Ecuador to the UK—have detailed workforce issues in banana and horticultural sectors, Australia’s banana industry remains under-researched. Existing work has largely focused on visa segmentation and labour policy [3,20,57], lacking focus on worker motivations or socio-demographic influences. Furthermore, agricultural job satisfaction research often generalizes across farming sectors, overlooking the specific challenges of seasonal and labour-intensive industries like Queensland’s banana farming. This study addresses that gap by empirically investigating the perceptions of banana industry workers in urban and regional Queensland, using statistical methods to identify factors affecting motivation, satisfaction, and retention. The aim is to generate data-driven insights to support workforce development, improve working conditions, and promote long-term sustainability in the sector.

Taken together, the literature reviewed above indicates that labour attraction, satisfaction, and retention in agriculture are shaped by an interaction of economic incentives, behavioural perceptions, and contextual work conditions rather than by isolated factors. Economic psychology and behavioural economics emphasise how fairness perceptions, aspiration gaps, and bounded rationality influence job-related decisions beyond purely wage-based considerations. At the same time, theories such as the Theory of Planned Behaviour and signalling theory highlight how attitudes, perceived norms, and organisational signals shape job choice and motivation. These perspectives collectively suggest that worker decisions in labour-intensive industries are driven by both objective job attributes and subjective interpretations of those attributes.

Drawing on this synthesis, the present study conceptualises workforce retention as a behavioural outcome influenced by job-related inputs (e.g., training opportunities, salary equity, and ergonomic conditions) and mediated by workers’ perceptions and job satisfaction. In this framework, ergonomic work design and organisational practices function as signals that shape employee attitudes, while behavioural biases and expectations influence how these signals are interpreted. Job satisfaction therefore operates as a central mechanism linking workplace conditions to retention outcomes.

**Fig 1:**
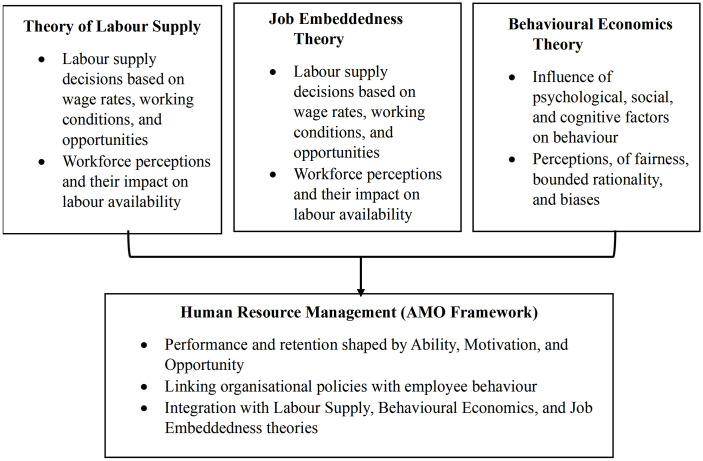
Integrated Theoretical Framework for Workforce Attraction, Satisfaction, and Retention.

Based on this integrated perspective, a unified conceptual framework is proposed (Fig 1), which links economic incentives, behavioural perceptions, and workplace conditions to labour satisfaction and retention in the Australian banana industry. The framework guides the selection of independent variables (training and development, ergonomic workplace design, salary equity, and growth opportunities), mediating constructs (job satisfaction and perception), and dependent outcomes (retention intention and workforce stability). This approach allows the study to empirically examine how behavioural and economic mechanisms jointly shape labour dynamics in a sector characterised by physical intensity and employment volatility.

## 3. Theoretical foundation

This study is grounded in three interrelated theoretical frameworks: the Theory of Labour Supply, the Job Embeddedness Theory, and the Behavioural Economics Theory. Together, these frameworks offer a comprehensive lens to understand how labour force perceptions shape job satisfaction and retention in Australia’s banana industry, a key component of the horticultural sector.

### 3.1. Theory of labour supply

The Theory of Labour Supply explains individuals’ decisions to offer their labour based on wage rates, working conditions, preferences, and alternative opportunities [58,59]. Within this study, Labour Supply Theory is used to explain how economic incentives—such as salary adequacy, job stability, and growth opportunities—influence workers’ evaluation of job attractiveness and their decision to remain in or exit the banana industry. In the banana industry context, worker availability is shaped not only by monetary rewards but also by job satisfaction and perceived industry value.

### 3.2. Job embeddedness theory

The Job Embeddedness Theory offers a broader understanding of retention by focusing on why people stay in jobs, emphasising links, fit, and sacrifice [60–63]. In this study, Job Embeddedness Theory is applied to capture the social, relational, and contextual factors influencing labour retention, particularly in regional banana production settings. It accounts for community ties, lifestyle alignment, and perceived losses if one leaves, which are particularly relevant in regional banana production contexts where employment decisions are closely intertwined with place-based and social considerations.

### 3.3. Behavioural economics theory

Behavioural Economics Theory complements this by challenging the notion of rational decision-making, focusing instead on how bounded rationality, fairness perceptions, and social norms influence employment choices [64–67]. In the context of this study, behavioural economics explains how workers’ subjective perceptions—such as perceived fairness, trust in employers, and optimism about future prospects—shape job satisfaction and perceived industry attractiveness beyond objective economic conditions. Liu et al. also highlight how job performance indirectly affects turnover through mediators like job embeddedness and movement desirability [68].

### 3.4. AMO framework (Human Resource Management Perspective)

These perspectives are unified through the Human Resource Management (HRM) framework, specifically the AMO model, which explains how retention and performance are influenced by ability, motivation, and opportunity [69]. In this study, the AMO framework serves as the operational mechanism that translates theoretical insights into measurable organisational practices. It links training and skill development to Ability, compensation and fairness to Motivation, and supportive work environments and participation to Opportunity, thereby providing a practical structure for variable selection and empirical analysis.

### 3.5. Theoretical integration and conceptual framework

Rather than treating these theories as independent explanations, this study integrates Labour Supply Theory, Job Embeddedness Theory, and Behavioural Economics through the AMO framework to form a unified conceptual foundation. Labour Supply Theory explains the economic rationale behind employment decisions, Job Embeddedness Theory captures the social and contextual forces that encourage retention, and Behavioural Economics accounts for perceptual and cognitive influences on job evaluation. The AMO framework synthesises these perspectives by operationalising them into human resource practices related to training (ability), incentives (motivation), and supportive and participatory work environments (opportunity).This integrated framework directly informs the selection of explanatory variables, the formulation of hypotheses, and the specification of the ordinal regression model used in this study. Table 1 summarises the alignment between the theoretical perspectives and the AMO framework, while Fig 1 visually presents the integrated conceptual model underpinning workforce attraction, satisfaction, and retention in the Australian banana industry.

**Table 1:** Summary of inter-theoretical alignment.

Theory	Key Focus	Link to AMO Framework
Labour Supply Theory	Economic incentives	Explains *why* motivation (e.g., salary, job security) matters
Behavioural Economics	Cognitive & emotional behaviour	Enriches *how* motivation and opportunity are perceived
Job Embeddedness Theory	Retention through social/psych ties	Expands the *contextual impact* of ability, motivation, and opportunity
AMO Framework (HRM)	HR-driven retention and performance	Integrates and operationalises all factors through HR practices

Fig 1 illustrates an integrated theoretical framework in which Labour Supply Theory, Job Embeddedness Theory, and Behavioural Economics Theory explain workforce decisions from economic, social, and behavioural perspectives, respectively. These theories are synthesised through the Human Resource Management (AMO) framework, which operationalises their insights by linking employee ability, motivation, and opportunity to performance and retention outcomes. Together, the framework shows how wages, working conditions, perceptions, and behavioural factors jointly shape labour availability, job attractiveness, and retention.

The integrated framework highlights that labour attraction and retention in the banana industry are not driven by economic incentives alone but emerge from the interaction of wages and working conditions, social and organisational embeddedness, and behavioural perceptions. By unifying these dimensions through the AMO framework, the study provides a coherent basis for analysing workforce dynamics and for designing targeted human resource strategies to enhance job attractiveness, satisfaction, and long-term retention.

## 4. Methodology

### 4.1. Objective, hypothesis, and research model

#### 4.1.1. Objective.

The primary objective of this study is to investigate the factors influencing job attraction, satisfaction, and retention in the banana industry in Queensland, Australia. Specifically, this research seeks to identify and analyse the relationships between various factors—such as work environment, salary, training opportunities, and promotion prospects—and their impact on employees’ perceptions and decisions related to job satisfaction and retention. Additionally, the study aims to explore how these factors collectively shape the attractiveness of the banana industry as a workplace.

#### 4.2.2. Hypothesis.

To bridge the theoretical framework and the empirical analysis, this study explicitly operationalises the proposed theories through a structured research model. Drawing on Labour Supply Theory, Job Embeddedness Theory, Behavioural Economics, and the AMO framework, the model conceptualises perceived job attractiveness of the banana industry as the primary dependent variable, measured on an ordinal scale. This outcome reflects workers’ overall evaluation of employment appeal, consistent with behavioural and economic perspectives that view job choice as a ranked, subjective decision rather than a binary outcome.

Within this framework, promotion and growth opportunities, training and development, supportive work environment, and salary levels are specified as key explanatory variables. These variables operationalise the economic incentives emphasised in Labour Supply Theory, the organisational and social attachments highlighted by Job Embeddedness Theory, and the perceptual and fairness considerations central to Behavioural Economics. The AMO framework provides the organising structure by linking training to Ability, salary and growth prospects to Motivation, and supportive work environments to Opportunity, thereby translating theoretical constructs into measurable workplace attributes. In addition, socio-demographic characteristics (gender, education level, and household status) are included as control variables to account for heterogeneity in preferences, constraints, and reference points across individuals.

The hypotheses guiding this study are as follows:

H_1_: The perceived attractiveness of employment in the banana industry significantly differs across ordinal levels.

Perceived job attractiveness is a subjective evaluation shaped by individual expectations, reference points, and trade-offs, and therefore is expected to vary significantly across ordinal levels rather than as a uniform assessment.

H_2_: Promotion and growth opportunities have a significant impact on the perceived attractiveness of the banana industry.

This hypothesis is grounded in Labour Supply Theory and Job Embeddedness Theory, which suggest that expectations of future advancement and career continuity enhance the perceived utility and organisational fit of a job, thereby increasing its attractiveness.

H_3_: Training and development opportunities significantly influence the attractiveness of the banana industry.

Consistent with the AMO framework and Labour Supply Theory, access to training enhances workers’ abilities and signals employer investment in human capital, which is expected to strengthen job appeal and motivation.

H_4_: A supportive work environment significantly contributes to the perceived attractiveness of the banana industry.

Drawing on Job Embeddedness Theory and signalling theory, a supportive workplace fosters social connections and conveys positive organisational values, thereby increasing employees’ attachment to and evaluation of the job.

H_5_: Salary levels significantly influence the attractiveness of the banana industry.

In line with Labour Supply Theory and Behavioural Economics, fair and competitive remuneration improves expected economic returns and fairness perceptions, which are central to workers’ job evaluations.

H_6_: Socio-demographic characteristics, including gender, education level, and household status, are significantly associated with perceived job attractiveness in the banana industry.

Based on behavioural economics and economic psychology, individual characteristics shape preferences, constraints, and reference points, leading to heterogeneous perceptions of job attractiveness across worker groups.

Hypotheses H2–H5 examine the drivers of job attractiveness primarily through satisfaction-related mechanisms, while H6 captures retention-related heterogeneity in job attractiveness arising from socio-demographic differences.

#### 4.2.3. Model.

Guided by this research model, an ordinal logistic regression is employed to estimate how these theoretically grounded factors influence the likelihood of higher levels of perceived job attractiveness. Perceived job attractiveness represents an integrative evaluation that simultaneously reflects job attraction, experienced satisfaction, and intention to remain. While job attraction, job satisfaction, and job retention are analytically distinct concepts, prior behavioural and labour research recognises that workers often form a global evaluative judgement that integrates these dimensions when assessing employment options. In this study, perceived attractiveness of the banana industry is conceptualised as such an integrative construct, reflecting (i) initial attraction to the industry, (ii) satisfaction with job conditions and experiences, and (iii) the perceived desirability of remaining in the industry. Accordingly, job attractiveness serves as a higher-order outcome that captures the combined influence of satisfaction- and retention-related considerations without modelling them as separate dependent variables. The following hypotheses are formulated to test the proposed relationships:

Let $Y_{i}$denote the perceived attractiveness of the banana industry for respondent i, measured on an ordinal scale with categories j=1,2,3,4,5. The proportional-odds (cumulative logit) model is specified as:


$$\text{log}(\frac{\text{Pr}\left(Y_{i}\leq j\right)}{\text{Pr}(Y_{i\;>}\;j)})=\;\alpha_{j}-(\beta_{\text{1}}{PGO}_{i}+\;\beta_{\text{2}}{TD}_{i}+\beta_{\text{3}}{SWE}_{i}+\;\beta_{\text{4}}{SAL}_{i}+\;\beta_{\text{5}}{GEN}_{i}+\;\beta_{\text{6}}{EDU}_{i}+\;\beta_{\text{7}}{HH}_{i}$$

j = 1,2,3,4

where:

• $\text{Pr}(Y_{i}\leq j)$ is the cumulative probability that respondent i reports perceived attractiveness of employment at level j or below,

• $\alpha_{j}$are the threshold (cut-point) parameters for each cumulative split,

• $PGO_{i}$ = promotion & growth opportunity,

• $TD_{i}$ = training & development,

• $SWE_{i}$ = supportive work environment,

• $SAL_{i}$ = salary,

• $GEN_{i}$ = gender, $EDU_{i}$= education, $HH_{i}$= household status,

• $\beta_{k}$ are regression coefficients.

A positive $\beta_{k}$increases the likelihood of being in a higher attractiveness category (i.e., shifts probability mass toward higher Y), while a negative $\beta_{k}$shifts probability toward lower categories, holding other variables constant.

Table 2 presents the mapping between the study’s theoretical frameworks, key conceptual constructs, corresponding empirical variables, and the associated research hypotheses used in the analytical model.

**Table 2:** Mapping of theoretical constructs to empirical variables.

Theory	Core Construct	Empirical Variable	Hypothesis
Labour Supply Theory	Economic incentives	Salary	H5
Labour Supply Theory	Future returns	Promotion & growth opportunity	H2
AMO Framework	Ability	Training & development	H3
AMO Framework	Opportunity	Supportive work environment	H4
Job Embeddedness Theory	Fit/ Links	Supportive work environment	H4
Behavioural Economics	Fairness & perception	Salary, perceived job attractiveness	H1, H5
Behavioural Economics	Preference heterogeneity	Gender, education, household status	H6

Socio-demographic variables, including gender, education level, and household status, are included as control variables to capture heterogeneity in preferences, constraints, and reference points. Behavioural economics and economic psychology suggest that individuals with different demographic and household characteristics evaluate job attributes differently due to varying opportunity costs, risk perceptions, and life-cycle considerations. Including these variables allows the model to isolate the effects of workplace attributes on perceived job attractiveness while accounting for individual-level differences.

### Survey instrument and pilot validation

The questionnaire was developed based on constructs widely used in the labour economics, human resource management, and job satisfaction literature, with items adapted and contextually modified to reflect the characteristics of the Australian banana industry. To ensure the reliability and validity of the measurement instrument, a pilot test was conducted in September 2024 using the Qualtrics platform, resulting in 37 completed responses. During the pilot phase, respondents were not only asked to complete the questionnaire but were also invited to provide feedback and suggest additional factors or questions that they believed should be considered. Insights from the pilot survey, together with preliminary reliability assessments, informed minor refinements, rewording, and clarification of several items. Following this pilot validation process, the questionnaire was finalised and subsequently administered in the main survey.

### Study sample

This study selected individuals aged 15–64 — the standard working-age population defined by the Australian Bureau of Statistics (ABS) and the OECD — to represent the labour force in Australia. A total of 1,202 respondents, current workers or potential workers, were surveyed to gather their perceptions of the Australian banana industry, a key sector within horticulture where labour is essential across different stages of the supply chain. The data were collected from 7^th^ November 2024–4^th^ December 2024 through a structured survey distributed by Dynata, a market research company. Quota sampling was employed to promote gender diversity and inclusivity among respondents. The final sample comprised 45% male and 45% female participants, with an additional 5% identifying as non-binary, third gender, or other, and 5% preferring not to disclose their gender. This quota design ensured balanced gender representation and supported inclusive analysis of preferences and behaviours. In terms of geographic distribution, the sample closely mirrored the population composition of the selected regions (see the supplementary material- Table A_1_), supporting the representativeness of the survey coverage. Brisbane accounted for 50.6% of respondents (compared with 47.5% of the population), followed by the Gold Coast (17.1%) and Sunshine Coast (12.2%), both slightly above their population shares. Key regional centres—including Toowoomba, Cairns, Townsville, Bundaberg, Mackay, and Rockhampton—were also proportionally represented, ensuring broad and balanced geographic coverage across Queensland. This study draws on an online panel sample that reflects the Queensland labour supply pool relevant to the banana industry, including both current workers and prospective workers. This design is intentional because the primary outcomes—perceived job attractiveness and related intentions—are perception-based constructs that directly shape labour supply decisions and recruitment feasibility. From a labour supply and signalling perspective, job attributes such as pay fairness, training, growth opportunities, and supportive work environments operate not only as internal retention mechanisms but also as external signals influencing whether individuals enter the sector. Accordingly, incorporating prospective workers provides policy-relevant evidence about the industry’s ability to attract new entrants alongside retaining existing labour. While perceptions may differ from lived experience, they are central to employment choice and therefore informative for workforce planning and HR interventions. All participants provided informed consent prior to taking part in the study. Consent was obtained in written form through the online questionnaire. Before accessing the survey items, respondents were presented with a detailed information sheet describing the study purpose, procedures, voluntary nature of participation, confidentiality, and data use. Participants were required to actively indicate their consent by selecting an “I agree to participate” option before they could proceed to the questionnaire. This electronic consent was automatically recorded by the survey platform and stored as part of the study dataset. The study did not involve minors; all participants were aged 18 years or older. Therefore, parental or guardian consent was not required. No waiver of consent was sought or granted, as informed consent was obtained from all participants in accordance with the approval of the Human Research Ethics Committee. The survey targeted a diverse range of respondents across Queensland, ensuring a representative sample from different demographic backgrounds, including gender, education, and household status. The respondents were asked to evaluate factors related to their job experiences using a 5-point Likert scale, ranging from “Strongly Disagree” (1) to “Strongly Agree” (5). The survey was designed to gather insights into their perceptions of job attraction, satisfaction, and retention factors.

### Questionnaire development

The questionnaire was carefully developed to capture the key elements influencing job attraction, satisfaction, and retention in the banana industry. It comprised several sections, each focused on one of the factors identified in the research objectives. The factors explored include:

1. **Perceived attractiveness of employment in the banana industry** (e.g., overall industry appeal, work-life balance, job security).
2. **Job satisfaction** (e.g., satisfaction with work environment, training opportunities, salary, and benefits).
3. **Job retention** (e.g., long-term commitment to the job, reasons for staying, and career advancement opportunities).

Each factor was measured on a 5-point Likert scale, where respondents were asked to rate their agreement with specific statements related to each factor. The scale allows for the measurement of ordinal data, enabling the study to explore the relationships between these factors and their impact on job satisfaction and retention. This methodology outlines the process for investigating job attraction, satisfaction, and retention factors in the banana industry using an ordinal regression model. The study utilizes a well-structured survey to gather data from 1,202 respondents across Australia, and the analysis using SPSS provides a comprehensive understanding of the key factors influencing employees’ perceptions of their jobs. The findings from this study will contribute valuable insights for industry stakeholders to improve job satisfaction and retention strategies.

### Ethical statement

This study was approved by the Human Research Ethics Committee of Central Queensland University, Australia, as part of the first author’s PhD project (Application Reference: 0000024996). All procedures involving human participants were conducted in accordance with the ethical standards of the institutional research committee. Informed consent was obtained from all individual participants included in the study.

### Data analysis

To explore the relationship between socio-demographic factors and perceptions of the banana industry’s attractiveness, the study initially employed Quantile-Quantile (Q-Q) plots as a diagnostic and exploratory visual tool. Q-Q plots were generated for key independent variables including gender, age, education level, household status, income before tax, and employment status using Stata software. The purpose of these plots was to examine the distributional characteristics of the independent variables and to identify potential outliers or extreme deviations during preliminary data screening, rather than to assess bivariate or linear relationships with the ordinal dependent variable. Q–Q plots were therefore used as an exploratory diagnostic to support data quality assessment prior to model estimation. As ordinal logistic regression does not require assumptions of normality or linearity, the Q–Q plots were not used to test model assumptions but to inform the overall modelling strategy by ensuring no severe distributional anomalies were present. The insights gained from Q-Q plotting informed the model development process, allowing for a more theoretically and statistically sound application of ordinal logistic regression in the next phase of analysis.

The data collected from the survey were analysed using SPSS (Statistical Package for the Social Sciences). An ordinal regression model was employed to examine the relationships between the independent variables (job attraction, satisfaction, and retention factors) and the dependent variable (job retention). Ordinal regression is suitable for this study as the dependent variable is ordinal in nature, represented by the 5-point Likert scale responses. The ordinal regression analysis was conducted to assess how well the independent variables predicted the likelihood of respondents being in a higher or lower category of job retention. The analysis involved examining the odds ratios for each factor, determining the strength and direction of their influence on job retention. The model also provided insights into the significance of various predictors, helping to identify the most critical factors influencing job satisfaction and retention in the banana industry.

## 5. Results

The findings indicate that perceived employment attractiveness in the banana industry is strongly shaped by ergonomic considerations, including safe equipment, appropriate task design, and physically supportive working conditions. Opportunities for promotion and growth—particularly when combined with training in ergonomic practices and workplace safety—can enhance motivation while reducing physical strain. Training and development initiatives that focus on posture, tool handling, and injury prevention emerge as critical mechanisms for improving ergonomic outcomes and sustaining workforce participation. A supportive work environment further reflects effective ergonomic design, where workflows, tools, and workspaces are aligned with human capabilities and limitations. Salary appears to function as a complementary signal of the value placed on workers’ health and safety, especially when remuneration is associated with reduced ergonomic risk. In addition, socio-demographic factors such as gender, education, and household status influence how ergonomic conditions are perceived and evaluated, with more educated workers and those with family responsibilities showing heightened sensitivity to physically demanding or unsafe environments. Collectively, these results underscore the central role of ergonomics in workforce management, linking job attractiveness to satisfaction, retention, and long-term sustainability in the horticultural sector. The data collected from the survey were analysed using SPSS (Statistical Package for the Social Sciences). An ordinal regression model was employed to examine the relationships between the independent variables (job attraction, satisfaction, and retention factors) and the dependent variable (job retention). Ordinal regression is suitable for this study as the dependent variable is ordinal in nature, represented by the 5-point Likert scale responses. The ordinal regression analysis was conducted to assess how well the independent variables predicted the likelihood of respondents being in a higher or lower category of job retention. The analysis involved examining the odds ratios for each factor, determining the strength and direction of their influence on job retention. The model also provided insights into the significance of various predictors, helping to identify the most critical factors influencing job satisfaction and retention in the banana industry.

Table 3 presents the responses of 1,202 participants, reflecting their perceptions of several factors related to employment in the banana industry. The majority of respondents consider the banana industry to be moderately attractive, with the largest group (34.0%) rating it as a 3, indicating a neutral to somewhat positive view. Fewer respondents rated it as highly unattractive, suggesting that the industry is generally perceived positively, though not overwhelmingly so. In terms of promotion and growth opportunities, 36.3% of respondents rated this factor as 4, indicating that most people perceive good opportunities for advancement within the industry. This is further supported by the fact that only 4.1% of respondents rated it as a 1, suggesting that promotion opportunities are not considered a significant limitation. Regarding training and development, 40.8% of respondents rated this aspect as 4, showing a positive view of the training and development opportunities within the industry. This is corroborated by the relatively low percentage (3.5%) of respondents rating it as 1, indicating dissatisfaction. When it comes to the supportive work environment, the majority of respondents (44.3%) rated it as a 5, suggesting that most people find the work environment to be highly supportive. Only 1.7% rated it as a 1, which implies that the vast majority of workers perceive the environment in a positive light. Salary is also viewed favourably by respondents, with 48.1% rating it as a 5, indicating that most people are satisfied with their compensation. Only a small percentage (1.5%) rated salary as a 1, showing minimal dissatisfaction with wage levels. Gender-wise, the survey demonstrates a relatively balanced distribution, with 51.1% of respondents identifying as male and 47.7% as female. The small number (1.2%) of other gender identities suggests that gender diversity in the workforce is limited but still present. Education levels are varied, with the largest group (32.3%) having an education rating of 7, likely indicating a higher level of education, such as a university degree. Only 0.2% of respondents rated it as 1 or 2, which suggests that very few participants have minimal education. This indicates that the workforce in the banana industry is relatively well-educated. Finally, in terms of household status, a slight majority (56.9%) of respondents fall into the second category, likely reflecting a more stable family situation, while 43.1% fall into the first category, possibly indicating less stability in their household status. Overall, the data reveals a generally positive view of working in the banana industry, with respondents particularly appreciating the salary, work environment, and opportunities for promotion and training. Education levels are high, and the workforce is somewhat balanced in terms of gender, with a tendency toward stable household situations.

**Table 3:** Descriptive analysis.

Descriptive analysis			
		N	Marginal Percentage
Perceived Attractiveness of Employment in the Banana Industry	1.00	111	9.2%
	2.00	315	26.2%
	3.00	409	34.0%
	4.00	264	22.0%
	5.00	67	5.6%
	6.00	36	3.0%
Promotion and growth opportunity	1.00	49	4.1%
	2.00	93	7.7%
	3.00	311	25.9%
	4.00	436	36.3%
	5.00	313	26.0%
Training and development	1.00	42	3.5%
	2.00	113	9.4%
	3.00	319	26.5%
	4.00	490	40.8%
	5.00	238	19.8%
Supportive work environment	1.00	21	1.7%
	2.00	31	2.6%
	3.00	153	12.7%
	4.00	465	38.7%
	5.00	532	44.3%
Salary	1.00	18	1.5%
	2.00	25	2.1%
	3.00	114	9.5%
	4.00	467	38.9%
	5.00	578	48.1%
Gender	1.00	573	47.7%
	2.00	614	51.1%
	3.00	15	1.2%
Education	1.00	3	0.2%
	2.00	2	0.2%
	3.00	53	4.4%
	4.00	235	19.6%
	5.00	201	16.7%
	6.00	160	13.3%
	7.00	388	32.3%
	8.00	58	4.8%
	9.00	102	8.5%
Household Status	1.00	518	43.1%
	2.00	684	56.9%
Valid		1202	100.0%
Missing		0	
Total		1202	

**Fig 2:**
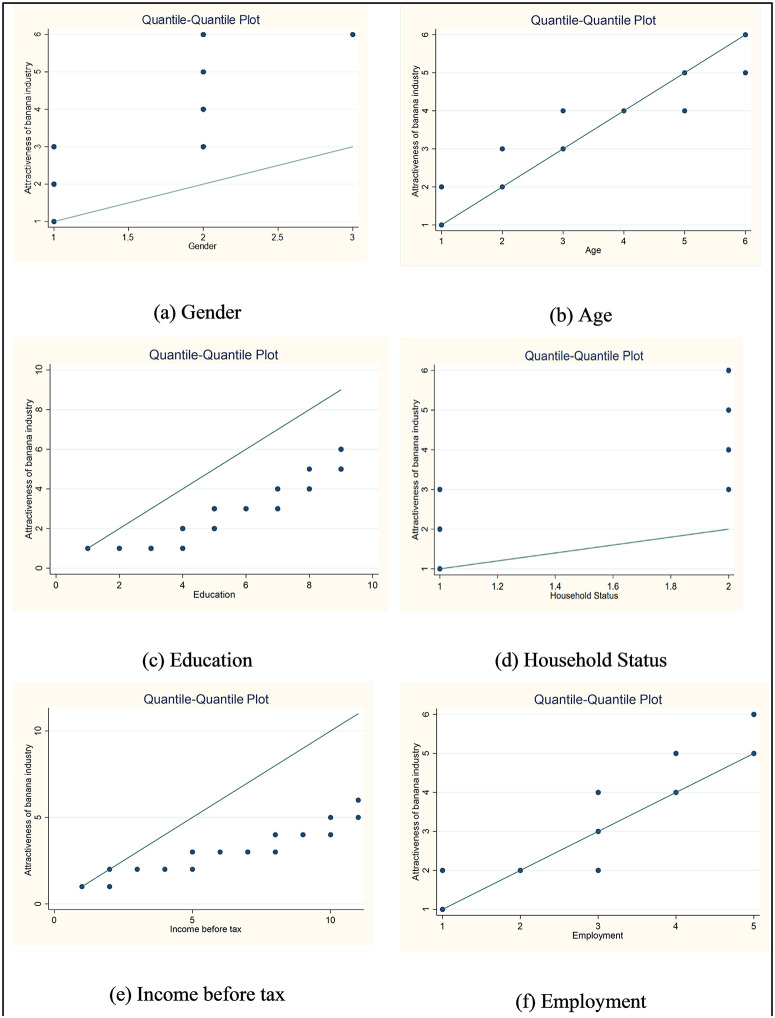
Quantile-Quantile plot of socio-economic and demographic variables.

Before presenting the regression results, the following Q-Q plots (Fig 2-a to 2-f) provide an initial visual analysis of how participants’ socio-demographic characteristics relate to their perception of the banana industry’s attractiveness (Fig 2). These figures serve as an important diagnostic step to detect distribution patterns and variations across groups, helping to uncover early signs of potential associations or inconsistencies. By comparing each variable’s observed quantiles to a theoretical normal distribution, the plots offer insight into whether perceptions vary systematically across demographic lines or display random scatter. These visual cues laid the groundwork for selecting variables for further analysis in the ordinal regression model, which follows this section.

Fig 2-a compares the distribution of gender (likely coded numerically: 1 = Male, 2 = Female, 3 = Other) with the perceived attractiveness of the banana industry (Fig 2-a). The data points deviate substantially from the reference line, indicating that the relationship between gender and perceived attractiveness does not follow a normal distribution. The vertical spread suggests that perceptions vary widely across gender categories, with no clear linear trend.

Moving from gender to age, Fig 2- b compares age categories (from 1 = 18–24 years to 6 = 60–below 65 years) with perceived attractiveness of the banana industry. The data points closely follow the reference line, indicating a relatively linear and positive relationship—older age groups generally perceive the banana industry as more attractive. Minor deviations at a few points suggest slight variability, but overall, the trend indicates that interest or appreciation for the industry tends to grow with age, possibly due to greater awareness of its economic and environmental significance.

Fig 2-c displays a generally linear trend between education level and the perceived attractiveness of the banana industry, indicating that as education increases, participants tend to view the industry more favourably. With education levels ranging from no formal education to postgraduate degrees, individuals with higher qualifications (e.g., Bachelor’s, Graduate, or Postgraduate degrees) are more likely to associate the banana industry with positive attributes. This pattern suggests a growing recognition of the industry’s potential for innovation, sustainability, and skilled job opportunities among more educated individuals.

In contrast, Fig 2-d compares household status (1 = Single/Separated/Divorced/Widowed, 2 = Married/Partner/Defacto) with perceived attractiveness of the banana industry. The plotted data points are widely dispersed and deviate from the reference line, indicating no clear linear or normal distribution between household status and industry perception. While both groups show a range of responses, the spread suggests that marital or partnership status does not consistently predict how attractive individuals perceive the banana industry to be.

Shifting focus to economic background, Fig 2-e illustrates the relationship between income before tax (coded from 1 = less than $20,000–11 = over $180,000) and perceived attractiveness of the banana industry. The data points show a fairly consistent upward trend that loosely follows the reference line, suggesting a positive association—as income increases, so does the perceived attractiveness of the banana industry. However, slight deviations from the line indicate that while the general trend is upward, perceptions still vary within income groups, possibly influenced by personal values or non-economic factors.

Fig 2-f compares employment status (coded from 1 = Student to 5 = Self-employed) with the perceived attractiveness of the banana industry. The data points align closely with the reference line, suggesting a roughly linear and positive relationship—those who are employed (particularly self-employed or in horticulture) tend to rate the industry as more attractive. The upward trend implies that direct engagement or autonomy in employment (like self-employment or working in horticulture) may positively influence perceptions of the banana industry, while students and unemployed individuals show lower attractiveness ratings.

Table 4 indicate that the final model provides a significantly better fit to the data compared to the intercept-only model. The −2 Log Likelihood for the final model is 2897.970, which is lower than the intercept-only model’s −2 Log Likelihood of 3085.339, suggesting that the inclusion of predictors improves the model’s explanatory power. The Chi-Square statistic of 187.369 with 54 degrees of freedom is significant, with a p-value of less than 0.001, indicating that the final model significantly improves the fit and that the predictors included in the model contribute meaningfully to explaining the outcome variable.

**Table 4:** Model fitting information.

Model Fitting Information				
Model	−2 Log Likelihood	Chi-Square	df	Sig.
Intercept Only	3085.339			
Final	2897.970	187.369	54	<.001
Link function: Logit.				

The Goodness-of-Fit statistics evaluate how well the model fits the observed data (Table 5). The two tests presented are the Pearson Chi-Square and the Deviance Chi-Square.

**Table 5:** Goodness of Fit.

Goodness-of-Fit			
	Chi-Square	df	Sig.
Pearson	3863.553	3791	.202
Deviance	2537.844	3791	1.000
Link function: Logit.			

### Pearson Chi-Square

The Pearson Chi-Square statistic is 3863.553 with 3791 degrees of freedom. The associated p-value is 0.202. A p-value greater than 0.05 indicates that the model provides a good fit to the data. In this case, the p-value suggests that there is no significant difference between the observed and expected values, implying that the model fits the data well. Essentially, the model is not significantly different from the observed data, indicating a good fit.

### Deviance Chi-Square

The Deviance statistic is 2537.844 with 3791 degrees of freedom, and the p-value is 1.000. A p-value of 1.000 means that the model fits the data perfectly, with no significant deviation between the observed and predicted values. In other words, the model explains the data well, and there is no evidence to suggest a poor fit.

**Fig 3:**
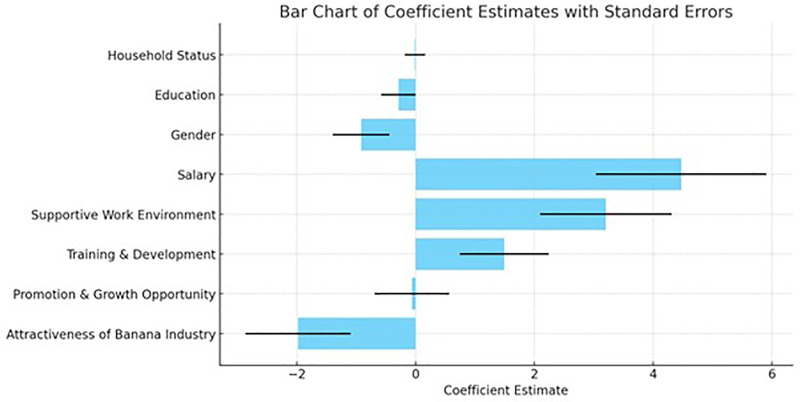
Coefficient estimates from the ordinal logistic regression model with standard errors.

Both the Pearson and Deviance Goodness-of-Fit tests indicate that the model fits the data well. The p-values for both tests (0.202 for Pearson and 1.000 for Deviance) suggest that there is no significant difference between the observed and expected values, supporting the conclusion that the model provides a good fit to the data (Fig 3).

Fig 3 illustrates the relative magnitude and direction of coefficient estimates from the ordinal logistic regression, along with their associated standard errors. Training and development, supportive work environment, and salary display the largest positive coefficients, indicating that these factors exert the strongest influence on perceived employment attractiveness in the banana industry. In contrast, promotion and growth opportunities show a comparatively small effect, while socio-demographic variables (gender, education, and household status) exhibit weaker and more variable associations, as reflected by smaller coefficients and wider error ranges. Overall, the figure visually reinforces that workplace-related and ergonomics-linked factors dominate over demographic characteristics in shaping perceptions of job attractiveness.

Table 5 represents the results of an ordered logistic regression model, where the dependent variable seems to be the *Perceived Attractiveness of Employment in the Banana Industry* with five ordered categories (1 –5). The streamlined table (Table 6) reports statistically significant and theoretically salient parameter estimates to emphasise the core findings, while the complete set of ordinal regression results, including all coefficients and reference categories, is presented in the supplementary document (Table A_1_) to ensure methodological transparency and reproducibility. The model includes various predictor variables, such as promotion and growth opportunity, training and development, supportive work environment, salary, gender, education, and household status. The purpose of this analysis is to examine the relationship between these predictors and the likelihood of a respondent rating the attractiveness of the banana industry at different levels (1 –5).

**Table 6:** Key determinants of perceived employment attractiveness in the banana industry (Ordinal Regression Model)*.*

Variable	Category/ Level	Estimate	Std. Error	Wald χ²	Sig.	95% CI
**Promotion and Growth Opportunity**	Level 1	−0.920	0.478	3.697	0.054†	[-1.857, 0.018]
**Training and Development**	Level 2	−1.029	0.403	6.526	0.011**	[-1.818, -0.239]
	Level 3	−0.502	0.234	4.611	0.032**	[-0.961, -0.044]
**Supportive Work Environment**	Level 3	0.554	0.258	4.628	0.031**	[0.049, 1.059]
	Level 4	0.367	0.164	5.039	0.025**	[0.047, 0.688]
**Salary**	Level 1	−2.322	1.201	3.740	0.053†	[-4.676, 0.031]
**Education Level**	Graduate Cert./Dip.	−0.831	0.358	5.373	0.020**	[-1.533, -0.128]
**Household Status**	Single/ Sep./ Div./ Wid.	−0.350	0.151	5.404	0.020**	[-0.645, -0.055]

*Note.* Entries report statistically significant and theoretically relevant estimates from an ordinal logistic regression with a logit link. Reference categories are omitted for clarity. † p < 0.10, ** p < 0.05. Full model estimates are reported in the supplementary document-Table A_1_.

### Threshold (Perceived Attractiveness of Employment in the Banana Industry)

These are the threshold estimates that show the cutoff points for different categories of the dependent variable. They represent the log odds of being in a higher category of *Perceived Attractiveness of Employment in the Banana Industry* relative to a baseline.

The threshold estimates indicate the cutoff points for different levels of attractiveness ratings in the banana industry. For responses rated “1” (least attractive), the coefficient of −1.982 with a significant p-value (0.025) suggests a lower likelihood of a response being in this category as the value increases. However, responses rated “2” showed no significant impact (p = 0.919). A positive and significant coefficient of 1.494 for responses rated “3” (p = 0.045) indicates an increased likelihood of being in this category. Similarly, responses rated “4” and “5” demonstrated strong positive impacts, with coefficients of 3.205 (p = 0.004) and 4.473 (p = 0.002), respectively, showing increased attractiveness perceptions in higher categories.

### Location variables

These include *Promotion and Growth Opportunity*, *Training and Development*, and *Supportive Work Environment*, all of which influence perceptions about the banana industry’s attractiveness.

### Promotion and Growth opportunity

The coefficient for Promotion and Growth Opportunity = 1 (p = 0.054) is marginally significant, suggesting that lower ratings of promotion and growth opportunities decrease the likelihood of higher attractiveness ratings. Promotion and Growth Opportunity = 2–5: None of these have significant p-values, suggesting they do not strongly influence the perceived attractiveness.

### Training and development

For Training and Development = 2 and Training and Development = 3, there is a significant negative relationship with attractiveness (p-values 0.011 and 0.032, respectively), suggesting that lower levels of training and development decrease attractiveness. Training and Development = 4 does not have a significant effect. Training and Development = 5 has no effect due to redundancy in the model.

### Supportive work environment

Supportive Work Environment = 3 and Supportive Work Environment = 4 are both significant (p-values 0.031 and 0.025), indicating that a supportive work environment increases the likelihood of a higher attractiveness rating. Other categories do not show significant relationships.

### Salary

The salary variable shows mixed results. A near-significant negative effect for Salary = 1 (p = 0.053) indicates that lower salary levels reduce the attractiveness of the industry. However, other salary levels ([2] – [4]) did not show significant relationships, suggesting an inconsistent effect on perceptions of attractiveness.

### Gender

Gender differences were not statistically significant in determining the attractiveness of the banana industry. While both male (Gender = 1) and female (Gender = 2) respondents showed positive coefficients, the p-values (0.068 and 0.146) suggest no clear distinctions based on gender.

### Education

Education levels had a marginally significant negative effect on attractiveness. Respondents with the highest level of education (Education = 1) were slightly less likely to rate the industry as attractive (p = 0.074). Additionally, Education = 8 showed a significant negative effect (p = 0.020), indicating that certain levels of education might reduce perceived attractiveness. Other education levels were not significant.

### Household Status

Household status emerged as a significant predictor, with respondents categorized under Household Status = 1 showing a significant negative effect (p = 0.020). This indicates that individuals in this category are less likely to rate the banana industry as attractive compared to others.

### Scale (Model-wide Effects)

This section assesses how the various predictor variables impact the *odds* of being in a higher category of the dependent variable across all levels.

- **Promotion and Growth Opportunity and Training and Development:** These factors continue to show marginal effects, but overall, their impact on the scale is not as significant as individual categories.
- **Salary:** A significant effect (p = 0.028) for Salary = 1 suggests that salary plays a more prominent role in determining attractiveness across all levels.
- **Gender and Education:** These variables do not have significant effects on the scale.

Among the identified determinants, training and development and supportive work environments demonstrate the most robust and consistent associations with perceived job attractiveness, highlighting ergonomics-oriented skill development and workplace design as central levers for workforce stability. In contrast, promotion opportunities and salary exhibit marginal effects, suggesting that while economic and career incentives matter, their influence may be conditional on how they interact with physical working conditions and perceived job sustainability. These weaker yet suggestive relationships should therefore be interpreted cautiously and viewed as indicative rather than definitive. Future research could explore these dynamics through longitudinal designs or mixed method approaches to better capture how financial and career incentives translate into long-term retention decisions under varying ergonomic conditions. Additionally, the selective significance of socio-demographic factors points to the need for more nuanced investigations into how personal circumstances shape sensitivity to physical work demands across different agricultural contexts.

**Fig 4:**
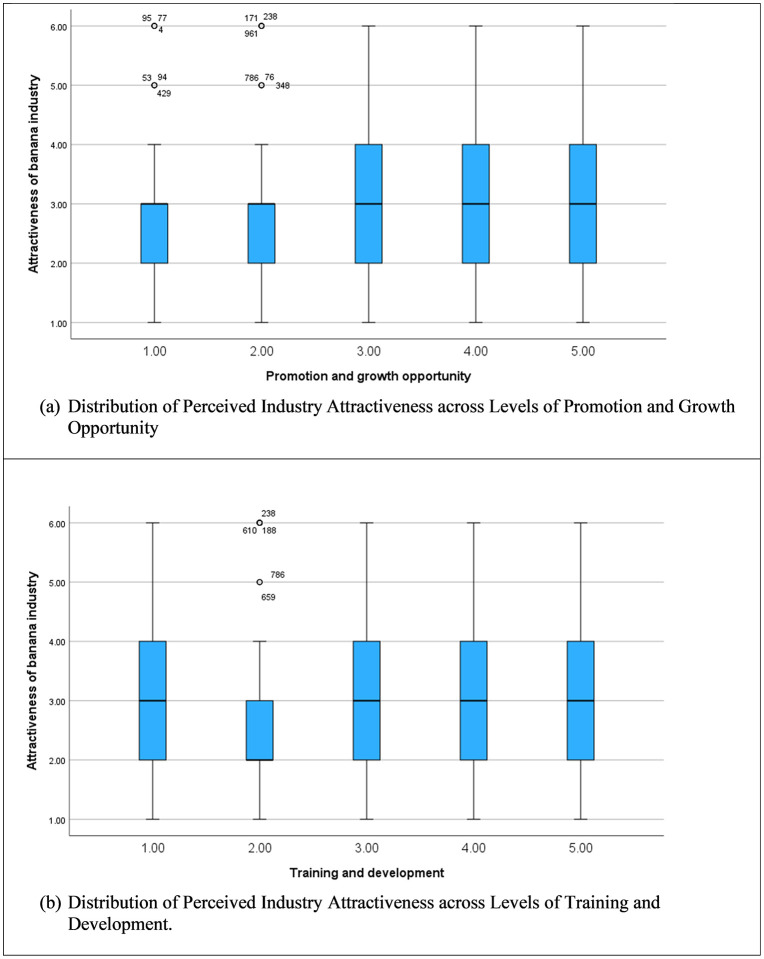
Variation in Perceived Employment Attractiveness by Promotion and Skill Development Opportunities.

Fig 4 (a) and (b) illustrate how perceived attractiveness of the banana industry varies across levels of promotion and growth opportunity and training and development. In both cases, median attractiveness scores increase as opportunity levels rise, indicating a generally positive association. The effect appears more pronounced and consistent for training and development, where higher levels are associated with a clearer upward shift in medians and a more stable interquartile range, suggesting broader agreement among respondents. Promotion and growth opportunities show a positive but more variable pattern, with greater dispersion and several high-value outliers at lower levels, indicating heterogeneous perceptions. Overall, the figures suggest that while both factors enhance perceived industry attractiveness, training and development exerts a stronger and more uniform influence than promotion and growth opportunities.

**Fig 5:**
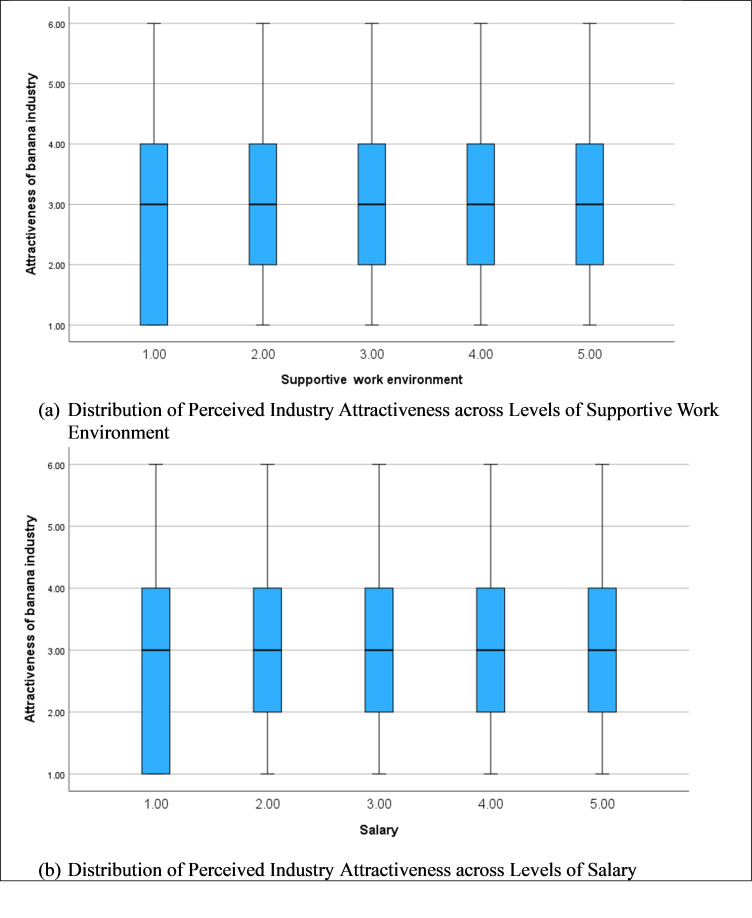
Variation in Perceived Employment Attractiveness by Supportive Work Environment and Salary.

Fig 5 (a) and (b) demonstrate how perceived attractiveness of the banana industry varies across levels of supportive work environment and salary. For supportive work environment, median attractiveness scores remain relatively stable around the mid-range but display a slightly upward shift and reduced dispersion at higher levels, suggesting that more supportive environments are associated with more consistently positive perceptions of industry attractiveness. In contrast, the salary plot reveals substantial overlap across levels, with similar medians and wide interquartile ranges, indicating greater heterogeneity in how pay influences perceptions. While higher salary levels do not uniformly raise perceived attractiveness, extreme values appear to matter more than incremental changes. Overall, the figures suggest that a supportive work environment exerts a steadier influence on perceived attractiveness than salary, which operates in a more variable and threshold-dependent manner.

Taken together, the workforce-related results indicate that job attractiveness in the Australian banana industry is driven more strongly by qualitative employment conditions than by purely economic incentives. Training and development and supportive work environments emerge as the most consistent and influential factors, reinforcing the importance of ergonomics-oriented skill development, safety, and workplace support in shaping positive workforce perceptions. Promotion opportunities and salary, while relevant, display more heterogeneous and threshold-dependent effects, suggesting that their impact depends on how they are embedded within broader working conditions. Overall, these findings highlight the need for workforce strategies that prioritise human capital development and supportive work environments to enhance attraction, satisfaction, and long-term retention in the horticultural sector.

**Fig 6:**
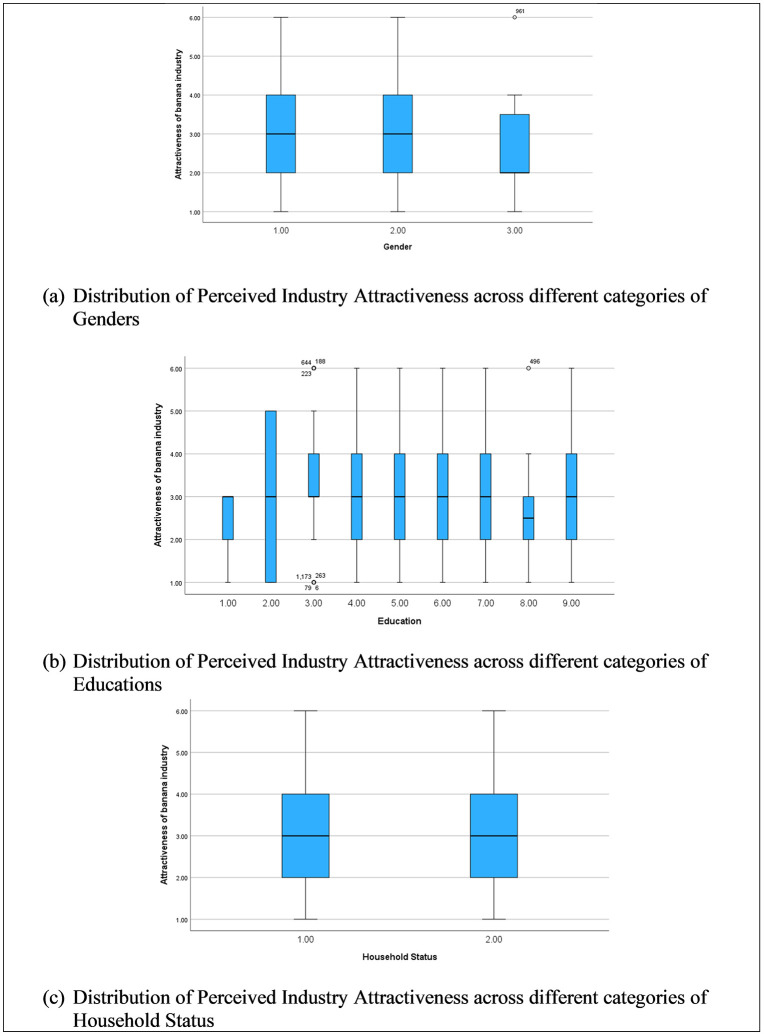
Variation in Perceived Employment Attractiveness by Genders, Education and Household status.

Fig 6 (a), (b) and (c) illustrate how perceived attractiveness of the banana industry varies across gender, education level, and household status. Overall, median attractiveness scores remain broadly similar across gender categories, with substantial overlap in interquartile ranges, indicating limited systematic gender-based differences and supporting the weak or non-significant gender effects observed in the regression analysis. In contrast, education shows greater variability, with some higher education levels displaying wider dispersion and slightly lower medians, suggesting more heterogeneous and, in some cases, more critical evaluations of industry attractiveness among highly educated respondents. Household status exhibits modest differences, with partnered respondents showing slightly more stable and positive perceptions compared to single or separated individuals, though distributions still overlap considerably. Collectively, these figures suggest that socio-demographic factors shape perceptions in nuanced and context-dependent ways, exerting a weaker and less uniform influence than core job-related factors.

### Hypothesis evaluation and empirical support

This section summarises the empirical evaluation of the proposed hypotheses, explicitly indicating the extent to which each is supported by the ordinal logistic regression results. Explicitly evaluating each hypothesis is important because it ensures transparency in linking theoretical expectations with empirical evidence.

Table 7 provides consistent support for all proposed hypotheses. Significant threshold effects confirm meaningful variation in perceived job attractiveness across ordinal levels (H_1_). Job-related factors—promotion opportunities, training and development, supportive work environments, and salary—each show statistically significant or marginally significant effects, underscoring their importance in shaping employment attractiveness in the banana industry (H_2_–H_5_). In addition, socio-demographic characteristics play a role, with both education level and household status significantly influencing perceptions of job attractiveness (H_6a_–H_6b_).

**Table 7:** Evaluation of research hypothesis and supported outcomes.

Hypothesis	Hypothesised Relationship	Empirical Evidence (Ordinal Logistic Regression Results)	Outcome
**H**_**1**_	The perceived attractiveness of employment in the banana industry significantly differs across ordinal levels	Several threshold parameters are statistically significant (p < 0.05), indicating meaningful variation across ordered categories of perceived job attractiveness	**Supported**
**H**_**2**_	Promotion and growth opportunities have a significant impact on perceived job attractiveness	Promotion and growth opportunity shows marginal significance at lower levels (p ≈ 0.05).	**Supported**
**H**_**3**_	Training and development opportunities significantly influence perceived job attractiveness	Training and development levels exhibit statistically significant effects at intermediate categories (p < 0.05), indicating a robust association with job attractiveness	**Supported**
**H**_**4**_	A supportive work environment significantly contributes to perceived job attractiveness	Supportive work environment shows positive and statistically significant effects at Levels 3 and 4 (p < 0.05), confirming its influence on job attractiveness	**Supported**
**H**_**5**_	Salary levels significantly influence perceived job attractiveness	Salary demonstrates marginal significance at lower levels (p ≈ 0.05).	**Supported**
**H**_**6a**_	Education level is significantly associated with perceived job attractiveness	Holding a Graduate Certificate or Graduate Diploma is associated with a statistically significant effect on perceived job attractiveness (p < 0.05)	**Supported**
**H**_**6b**_	Household status is significantly associated with perceived job attractiveness	Single/Separated/Divorced/Widowed respondents report significantly lower perceived job attractiveness compared to partnered respondents (p < 0.05)	**Supported**

## 6. Discussion of key insights

Overall, the findings indicate that the attractiveness of the Australian banana industry as a workplace is primarily shaped by different job characteristics, with training and development and supportive work environments emerging as the most robust and substantively important predictors, while salary, location, and demographic effects show more nuanced and conditional influences. The attractiveness of the Australian banana industry as a workplace is shaped by both work-related and demographic factors, many of which are closely tied to ergonomic principles affecting workers’ physical and psychological well-being. Key elements such as training and development, supportive work environments, and fair salaries contribute not only to positive perceptions of the industry but also highlight the role of ergonomics in creating healthier, safer, and more productive workplaces. This aligns with Lamm et al., who emphasize the importance of incorporating ergonomic awareness into education to address workforce challenges in horticulture [70]. Similarly, Meyer et al. noted that supportive environments and clear career pathways are crucial, especially for younger and diverse workers who value ergonomic safety and comfort [71]. Salary equity, a significant influence in this study, reflects the need to fairly compensate roles that prioritize ergonomics to reduce injury risk and enhance efficiency, echoing Waliczek et al. [72]. Furthermore, VanDerZanden and Reinert emphasize the role of work ethic and skills, often nurtured in ergonomically supportive environments, in boosting workforce sustainability [73]. By fostering healthier workplaces and stable employment, the industry can minimize labour-related disruptions that often lead to on-farm food losses and post-harvest waste.

While these job-related factors demonstrate strong and consistent associations with perceived industry attractiveness, demographic characteristics exhibit more subtle and context-dependent effects that warrant cautious interpretation. Demographic characteristics, such as education and gender, though not dominant predictors, revealed subtle patterns. Higher-educated individuals may perceive limited career growth, while women may assess safety, inclusion, or flexibility differently—though these differences were not significant across all thresholds. These findings resonate with Meyerding & Lehberger, who found no overarching gender-based differences in job satisfaction, but variability in the importance of job attributes [34]. Similarly, Scott et al. and Meyerding affirm that intersectional experiences shape workforce perceptions, underscoring the importance of inclusive, demographic-sensitive strategies for recruitment and retention in horticulture [34,74]. Inclusive workforce policies not only improve social equity but also help ensure a reliable labour supply, which is critical to preventing crop losses and the associated food waste during harvest and packing.

Salary-related effects, while influential, appear to operate in a non-linear and threshold-dependent manner, suggesting that remuneration alone does not uniformly shape perceptions of industry attractiveness. The role of salary in shaping perceptions varied depending on its relative level. While moderate salaries may not significantly influence opinions, extreme salary levels—either very low or very high—had a stronger impact. Low compensation continues to be a critical deterrent, as noted by Smeal, especially when compared to other job options [75]. Even with non-monetary benefits such as job satisfaction and job security [76], very low salaries strongly shape negative perceptions. Conversely, high salaries—though rare—may offset concerns about job risks or instability [77]. Salary can also signal role complexity and value in diversified horticultural careers [71,78,79]. Although the sector offers strong job prospects—with five to ten roles per graduate without competitive pay, it risks failing to attract top talent [75]. Technological progress and focus on worker welfare may raise compensation, improving the sector’s attractiveness for recruitment and retention [77]. These findings suggest that future research should examine how salary interacts with different job characteristics, job security, and career sustainability over time, particularly using longitudinal or mixed-method designs. Fair and competitive wages also reduce the likelihood of labour shortages that can delay harvesting and contribute to significant banana waste.

Location-based differences further highlight the contextual nature of workforce perceptions, indicating that industry attractiveness cannot be understood independently of regional labour market conditions. The survey results reveal that location influences perceptions of industry attractiveness, but not uniformly across all worker groups. This is supported by Miles et al., who highlight the challenges of attracting and retaining skilled workers in regional and rural Australia, particularly in agriculture [80]. They emphasize that career prospects, family considerations, and income significantly shape individual employment decisions. For example, regional workers may prioritize job stability and community integration, while urban workers may be more influenced by alternative employment opportunities and lifestyle factors. This mirrors the survey’s finding that perceptions vary across locations. Furthermore, the survey found no universal predictors of industry attractiveness across all groups, with some factors becoming significant only at higher perception thresholds (e.g., high Likert scale ratings). This aligns with Miles et al.’s argument that workforce strategies must be tailored to specific regional and professional contexts, as generic approaches are often ineffective [80]. The contribution of immigrant workers in addressing labour shortages and enhancing innovation in agriculture, as noted by Collins et al., further validates the need for segment-specific strategies [81]. Immigrant workers may value job security and social integration differently from local workers, contributing to the observed variability in perceptions. Amarakoon and Colley support this by advocating for nuanced employer branding and a balanced approach to formal and informal HR practices, adapted to local contexts [82]. Becker et al. further emphasize the importance of community-business collaboration in regional areas, reinforcing the survey’s conclusion that effective workforce engagement must address broader social integration, not just employment incentives [83]. Such tailored workforce strategies are essential to reduce harvest delays and post-harvest losses, thereby directly lowering food waste in the banana supply chain. Together, these nuanced patterns indicate the need for segment-specific workforce strategies and motivate future research into how regional, demographic, and ergonomic factors jointly shape labour attraction and retention decisions. Overall, these findings provide a nuanced and data-driven understanding of what drives workforce perceptions in the banana industry. They point to the need for strategic investment in human capital development, workplace culture, and compensation equity to build a more attractive, sustainable labour environment.

## 7. Conclusion and future research recommendations

It is evident that multiple interrelated factors, many tied to ergonomic principles, shape workforce perceptions in the Australian banana industry, with significant implications for job motivation, satisfaction, and retention. Workforce instability and high turnover not only disrupt production schedules but also increase the risk of crop losses, harvest delays, and post-harvest handling inefficiencies that contribute directly to food waste. Strengthening labour stability is therefore essential for minimising on-farm and supply-chain wastage. Robust empirical insights are needed to pinpoint the key drivers of workforce attraction and retention, enabling evidence-based strategies that effectively reduce both labour turnover and the associated food losses. This study provides empirical insights into how ergonomics-related variables—such as training and development focused on safe and efficient work practices, a supportive work environment designed to reduce physical strain, and competitive salary reflecting the value of ergonomic improvements—enhance the perceived attractiveness of jobs in this sector. By quantitatively linking these factors to job motivation, satisfaction, and retention, it fills a critical knowledge gap in horticultural labour research, where ergonomic considerations have often been acknowledged but rarely measured and analysed in a systematic, evidence-based way. While demographic factors like education and gender showed context-specific effects, these nuances highlight the need to consider ergonomic needs that vary across different worker groups. The findings suggest that the banana industry must implement targeted, ergonomically informed workforce strategies to address labour shortages and improve retention. Moreover, the variability in how different factors influence job attractiveness across rating levels underscores that workers assess job quality and industry appeal through diverse ergonomic, personal, and professional lenses. This calls for dynamic human resource approaches that integrate ergonomic considerations tailored to individual needs, thereby promoting long-term workforce engagement and well-being. A key limitation of this study is that it relies on perception-based responses from an online panel including both current and prospective workers, as accessing on-farm banana industry employees for survey participation is particularly challenging due to their demanding and time-intensive work schedules, although these perceptions remain highly relevant for labour supply and recruitment decisions. Despite this limitation, the contribution of the study exceeds this constraint by providing robust, policy-relevant insights into labour supply perceptions from a broad pool of current and prospective workers, thereby informing recruitment and retention strategies in a sector where direct access to on-farm employees is inherently limited. Given the scope and limitations of this study, several avenues for future research are recommended:

1. **Longitudinal Analysis**: Future studies should adopt a longitudinal approach to examine how workers’ perceptions evolve over time, particularly in response to industry policy shifts or economic changes.
2. **Comparative Industry Studies**: Expanding the research to compare the banana industry with other horticultural or agricultural sectors could reveal whether identified motivational and satisfaction factors are industry-specific or sector-wide.
3. **Migrant Labour Perspectives**: Given the industry’s reliance on temporary migrant workers, further research should specifically explore their unique experiences, motivations, and retention challenges under different visa arrangements.
4. **Intersectionality and Inclusion**: Future work should delve deeper into how intersecting identities (e.g., gender, ethnicity, visa status, education level) influence job satisfaction and workforce engagement.
5. **Organizational Practices and Leadership Styles**: Research into how management practices, leadership, and organizational culture affect employee satisfaction and turnover could provide actionable recommendations for industry leaders.

By addressing these gaps, future research can deepen our understanding of labour dynamics in horticulture and contribute to the development of more resilient, equitable, and attractive work environments in Australia’s agricultural landscape.

## Supporting information

S1 File Full Ordinal Logistic Regression Output for Perceived Employment Attractiveness. (PDF)
