# Supplementary material for: Aligning labour force perceptions to job attraction, satisfaction and retention strategies in horticulture: A data-driven empirical study for Australian banana industry
Source: PLoS One. 2026 Apr 20;21(4):e0344799. doi: 10.1371/journal.pone.0344799 (PMC13095013; doi:10.1371/journal.pone.0344799)
Supplement: S1 File — (PDF) [file pone.0344799.s001.pdf]

Supplementary material

S1 Table

Full Ordinal Logistic Regression Output for Perceived Employment Attractiveness

Variable	Estimate	Std. Error	Wald χ^2	df	Sig.	95% CI (Lower)	95% CI (Upper)
**Thresholds**							
Perceived Employment Attractiveness = 1	-1.982	0.882	5.048	1	0.025	-3.712	-0.253
Perceived Employment Attractiveness = 2	-0.064	0.631	0.010	1	0.919	-1.300	1.172
Perceived Employment Attractiveness = 3	1.494	0.747	4.002	1	0.045	0.030	2.957
Perceived Employment Attractiveness = 4	3.205	1.106	8.393	1	0.004	1.037	5.374
Perceived Employment Attractiveness = 5	4.473	1.437	9.691	1	0.002	1.657	7.290
**Promotion and Growth Opportunity**							
Level 1	-0.920	0.478	3.697	1	0.054	-1.857	0.018
Level 2	-0.290	0.290	1.001	1	0.317	-0.858	0.278
Level 3	-0.012	0.172	0.005	1	0.943	-0.350	0.325
Level 4	-0.181	0.153	1.391	1	0.238	-0.481	0.119
Level 5	Ref.	—	—	—	—	—	—
**Training and Development**							
Level 1	0.032	0.429	0.006	1	0.940	-0.809	0.873
Level 2	-1.029	0.403	6.526	1	0.011	-1.818	-0.239
Level 3	-0.502	0.234	4.611	1	0.032	-0.961	-0.044
Level 4	-0.204	0.161	1.606	1	0.205	-0.519	0.111
Level 5	Ref.	—	—	—	—	—	—
**Supportive Work Environment**							
Level 1	1.123	0.762	2.176	1	0.140	-0.369	2.616
Level 2	0.947	0.556	2.902	1	0.088	-0.142	2.036
Level 3	0.554	0.258	4.628	1	0.031	0.049	1.059
Level 4	0.367	0.164	5.039	1	0.025	0.047	0.688
Level 5	Ref.	—	—	—	—	—	—
**Salary**							
Level 1	-2.322	1.201	3.740	1	0.053	-4.676	0.031
Level 2	0.666	0.482	1.912	1	0.167	-0.278	1.611
Level 3	0.093	0.227	0.169	1	0.681	-0.351	0.538

Variable	Estimate	Std. Error	Wald χ^2	df	Sig.	95% CI (Lower)	95% CI (Upper)
Level 4	-0.013	0.116	0.012	1	0.913	-0.240	0.215
Level 5	Ref.	—	—	—	—	—	—
**Gender**							
Male	1.239	0.680	3.322	1	0.068	-0.093	2.571
Female	0.930	0.639	2.114	1	0.146	-0.323	2.183
Non-binary / Third gender / Others	Ref.	—	—	—	—	—	—
**Education Level**							
No formal education	-1.201	0.672	3.194	1	0.074	-2.519	0.116
Primary education	-0.288	4.158	0.005	1	0.945	-8.437	7.861
Secondary education (Year 10 or below)	0.179	0.318	0.315	1	0.575	-0.445	0.802
Senior secondary education (Year 11–12)	-0.296	0.227	1.700	1	0.192	-0.742	0.149
Certificate I–IV	0.001	0.225	0.000	1	0.995	-0.439	0.442
Diploma / Advanced Diploma	-0.032	0.246	0.017	1	0.898	-0.514	0.451
Bachelor's degree	-0.175	0.208	0.708	1	0.400	-0.583	0.233
Graduate Certificate / Graduate Diploma	-0.831	0.358	5.373	1	0.020	-1.533	-0.128
Postgraduate degree	Ref.	—	—	—	—	—	—
**Household Status**							
Single / Separated / Divorced / Widowed	-0.350	0.151	5.404	1	0.020	-0.645	-0.055
Married / Partner / De facto	Ref.	—	—	—	—	—	—

Table S1 reports the full ordinal logistic regression output, including non-significant coefficients and reference categories, to ensure transparency and replicability.
